# Effects of a Virtual Interactive System (People’s War Against Pandemic) on Emergency Preparedness for Major Emerging Infectious Diseases Among Adolescents: Randomized Controlled Trial

**DOI:** 10.2196/67595

**Published:** 2026-10-26

**Authors:** Wei Wei, Yue Luo, Min Tian, Longsheng Xie, Huan Li, Weili Hu, Yanhua Chen

**Affiliations:** 1 Center for Infectious Diseases, West China Hospital, Sichuan University, Chengdu, China; 2 School of Nursing, Southwest Medical University, Luzhou, China; 3 Department of Cardiology, West China Hospital of Sichuan University, Chengdu, China; 4 Department of Bone and Joint Surgery, The Affiliated Hospital of Southwest Medical University, Luzhou, China; 5 Department of Nephrology, The Affiliated Hospital of Southwest Medical University, Luzhou, China; 6 College of Humanities and Management, Southwest Medical University, Luzhou, China; 7 Department of Nursing, The Affiliated Hospital of Southwest Medical University, 25 Taiping Street, Luzhou, China, 86 18982765016

**Keywords:** major emerging infectious diseases, adolescents, emergency preparedness, virtual interactive, randomized controlled trial

## Abstract

**Background:**

The epidemiological situation of major emerging infectious diseases (MEIDs) continues to be severe. This requires each country and region to be vigilant, strengthen cooperation, and take effective prevention and response measures. This highlights the public’s demand for education on health and safety measures. Virtual interactive systems have played a critical role in many fields. Consequently, we designed and developed a virtual interactive system (People’s War Against Pandemic) aimed at training the public to respond to MEID outbreaks.

**Objective:**

This study aimed to evaluate the efficacy of a novel storyline-driven virtual interactive system (People’s War Against Pandemic) in enhancing emergency preparedness and infectious disease health literacy among adolescents.

**Methods:**

A single-blind (outcome assessors blinded), 2-arm, parallel-group randomized controlled trial was conducted in a secondary vocational school from October 2023 to December 2023 in Luzhou, Sichuan. A total of 120 adolescents were recruited through on-site recruitment and were randomly assigned to the intervention group (n=60) and the control group (n=60). The intervention group was trained using a virtual interactive system and traditional lectures, whereas the control group received only traditional lectures. Self-reported questionnaires assessing emergency preparedness for MEIDs and infectious disease health literacy were used to evaluate the effect of the virtual interactive system before and after the intervention. Repeated-measures ANOVA was used to scrutinize variations in outcome indicators across time points within and between groups.

**Results:**

The results showed that emergency preparedness and infectious disease health literacy scores improved in both the control and intervention groups. Additionally, emergency preparedness and infectious disease health literacy scores in the intervention group were higher than those in the control group (*P*<.001). A repeated-measures ANOVA revealed significant group, time, and group × time interaction effects in emergency preparedness and infectious disease health literacy scores.

**Conclusions:**

The virtual interactive system was effective in improving adolescents’ self-reported emergency preparedness and infectious disease health literacy in the face of MEIDs.

## Introduction

### Background

With the deepening development of globalization, increased population mobility, accelerated urbanization, and changes in the ecological environment, the frequency, scope, and impact of major emerging infectious diseases (MEIDs) in the world have increased in recent years [1]. MEIDs such as SARS, H1N1 influenza, Middle East respiratory syndrome (MERS), Ebola virus disease (EVD), and COVID-19 have posed significant threats to human health and social stability [2]. Research has indicated that the world will continue to face the growing and increasingly severe challenges posed by MEIDs [3]. Therefore, global preparedness for MEIDs is urgently needed, requiring heightened vigilance, strengthened cooperation, and effective preventive and response measures from every country and region.

Due to their distinctive physiological and psychological characteristics, adolescents exhibit more complex susceptibility to and transmissibility of MEIDs compared with adults, highlighting their vulnerability in public health emergency preparedness [4]. Adolescents spend most of their time in schools characterized by large populations and complex hierarchical structures, alongside frequent collective activities and numerous public spaces on campus. If there is a sudden outbreak of MEIDs, the spread rate is often extremely rapid. It seriously affects the progress of teaching, disrupts the orderly daily management of the campus, and potentially poses serious threats to the lives and safety of individuals within the school [5]. Meanwhile, it may cause widespread psychological panic and social disorder. Currently, the emergency preparedness among adolescents in many countries and regions remains deficient, which is mainly manifested by the lack of disease prevention knowledge, weak response capabilities in emergency situations, and insufficient protective measures and coping resources, which aggravate the threat of MEIDs to adolescents [6]. Therefore, enhancing the emergency preparedness among adolescents for MEIDs is a crucial task in safeguarding the health and social stability of adolescents. It is also an essential condition for achieving the Sustainable Development Goals related to health and well-being [7].

At present, education on emergency preparedness for adolescents is mainly based on traditional classroom teaching, brochures, and lectures [8]. However, surveys indicate that these methods often lack interest, interaction, and practicality, and thus struggle to attract the interest of adolescents [9]. The effectiveness of this education is poor, and the ability of adolescents to respond to MEIDs could not be effectively improved [9,10]. Additionally, the uneven distribution of educational resources means that adolescents in remote and impoverished areas often cannot access high-quality educational services, resulting in problems of educational equity [11].

In recent years, the rapid development of the information technology industry has continuously highlighted the application advantages of the virtual interactive training model, opening new avenues for enhancing emergency preparedness. Virtual interactive technology has been used for mass casualty education and military emergency training as part of urban or national emergency preparedness protocols [12,13] and to promote physical activity to reduce stress [14-16]. Notably, the health care industry has actively carried out virtual interactive training in emergency preparedness for MEIDs [17-19], demonstrating its efficacy in improving the emergency response capabilities of nursing students, medical workers, and emergency management personnel. This is supported by a study within the National Health Service (NHS) in the United Kingdom, which found that virtual reality (VR) training significantly improved the performance of frontline health care workers in infection control measures and personal protective equipment usage during the COVID-19 pandemic compared to traditional training methods [20]. Similarly, a systematic review and meta-analysis confirmed the positive impact of virtual experiments on learning effectiveness across various educational contexts [21]. However, a significant research gap remains. Previous interventions have primarily targeted professional populations within the health care or military sectors. There is a paucity of studies focusing on adolescents in general educational settings. Moreover, existing programs for the public often lack a comprehensive and engaging design that integrates knowledge acquisition, decision-making training, and behavioral simulation into an immersive, storyline-driven experience [22]. This limits their potential to effectively captivate and educate the adolescent demographic.

To fill this gap and better equip adolescents to cope with outbreaks of MEIDs, we designed and developed a virtual interactive system, “People’s War Against Pandemic” [23]. This system allows adolescents to learn essential knowledge and response skills through simulated outbreak scenarios within a narrative framework. This study aimed to test the effectiveness of this virtual interactive system in improving emergency preparedness for MEIDs among adolescents in Luzhou City, Southwest China. Here, we report the results of this study.

### Theoretical Framework: Embodied Cognition Theory

Embodied cognition theory posits that cognition arises from the dynamic interaction between the individual, the environment, and tools, and is grounded in sensory and bodily experiences, with perception serving as the basis for action [24]. In this study, the immersive, interactive, and imaginative features of the virtual system foster a deep sense of presence, enhancing emotional engagement. This affective response, mediated by presence, can restructure cognitive frameworks and ultimately promote behavioral change. Furthermore, the system supports the development of new body schemas [25], enabling the projection of real-world motor actions onto sensorimotor experiences within the virtual 3D environment [26]. Consequently, the activation of sensory and motor systems in this embodied context elicits bodily presence, linking physical actions to emotional and cognitive processing, which guides behavior and, in turn, enriches emotional experience, forming a cyclical feedback loop among emotion, cognition, and action (Figure 1).

**Figure 1.**
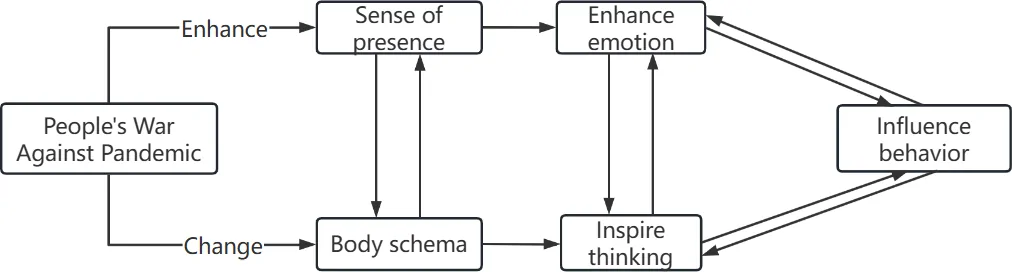
The realization path of virtual interactive system education under the theory of embodied cognition.

## Methods

### Study Design

This study was a single-blind, 2-arm, parallel-group randomized controlled trial (RCT). The CONSORT (Consolidated Standards of Reporting Trials) checklist was used when writing this report (Checklist 1).

### Sample Size Calculation

The sample size was calculated following a 2-mean test randomized controlled design: *n*_1_ = *n*_2_ = 2[(*t*_α/2_ + *t*_β_)*S* / δ]^2^. The primary indicator (emergency preparedness for MEIDs) was used to calculate the sample size for this study. A 2-sided test was used to set the test level (α=0.05) and the test efficiency (1 − β = 0.8); then *t*_α/2_=1.96 and *t*_β_=0.84. The SD (*S*=16.5) and the expected effect size (δ=9.5) were derived from an internal pilot study conducted by our research team prior to the main trial (n=12 adolescents recruited from the same target population). Based on these parameters, the required sample size was calculated as *n*_1_=*n*_2_=48. The total required sample size was 96 participants. Accounting for a 20% dropout rate, the sample size was calculated to be at least 60 participants in each group. It is important to note that this sample size is adequate for testing efficacy under controlled conditions but may not be representative of the entire adolescent population.

### Participants and Study Settings

This study used a single-center design, with participants recruited from a secondary vocational school in Luzhou, Sichuan. Thereafter, they were randomized into either a control group or an intervention group. This site was selected due to its accessibility and established collaboration, which facilitated the implementation and monitoring of the RCT. Although this approach enhanced the internal validity of the study by controlling for institutional-level confounding factors, it limits the generalizability of our findings to adolescents in other geographic, cultural, or educational settings. Therefore, the results should be interpreted as preliminary evidence of the intervention’s efficacy within this specific context.

The eligibility criteria for participation were as follows: (1) aged 10 to 19 years and (2) informed consent and voluntary participation in this study (participants below 18 y of age required the consent of the guardian). The exclusion criteria were as follows: (1) participants did not cooperate in completing the questionnaire and (2) participants were participating in or had participated in similar studies.

### Intervention Methods

#### Design of a Virtual Interactive System

The virtual interactive system was called People’s War Against Pandemic, and the interface diagram is shown in Figure 2. The background storyline is the outbreak of an MEID in a certain place, and participants need to identify and analyze the harmfulness and controllability of the pandemic’s development. At each stage of the pandemic’s spread, participants can act in each scene according to the development of the storyline and avoid infecting themselves and others through the correct selection of key interactive nodes. Participants can acquire knowledge and skills for the prevention and control of MEIDs, adjusting emergency-related emotions and changing behavioral tendencies. People’s War Against Pandemic has the following functional modules:

- Virtual interactive training module: There are 9 scenes and multiple tasks involved in the daily lives of teenagers.
- Knowledge corner module: It contains educational articles and videos on MEIDs.
- Intelligent evaluation module: It can help participants consolidate their knowledge of MEIDs.
- Forum community module: It provides users with a platform for communication.
- User management module: The educational information in the knowledge corner module can be edited, and data collection, integration, classification, and application can also be implemented.

Both groups received conventional lectures on MEIDs education, and the intervention group additionally received a lecture on the virtual interactive system developed by our team, resulting in a total intervention period of 4 weeks followed by a 2-week follow-up evaluation. The duration of the intervention and follow-up was designed to evaluate the initial efficacy and usability of the system; however, it did not allow for assessment of long-term knowledge retention or sustained behavioral change. The trial was conducted from October 2023 to December 2023.

**Figure 2.**
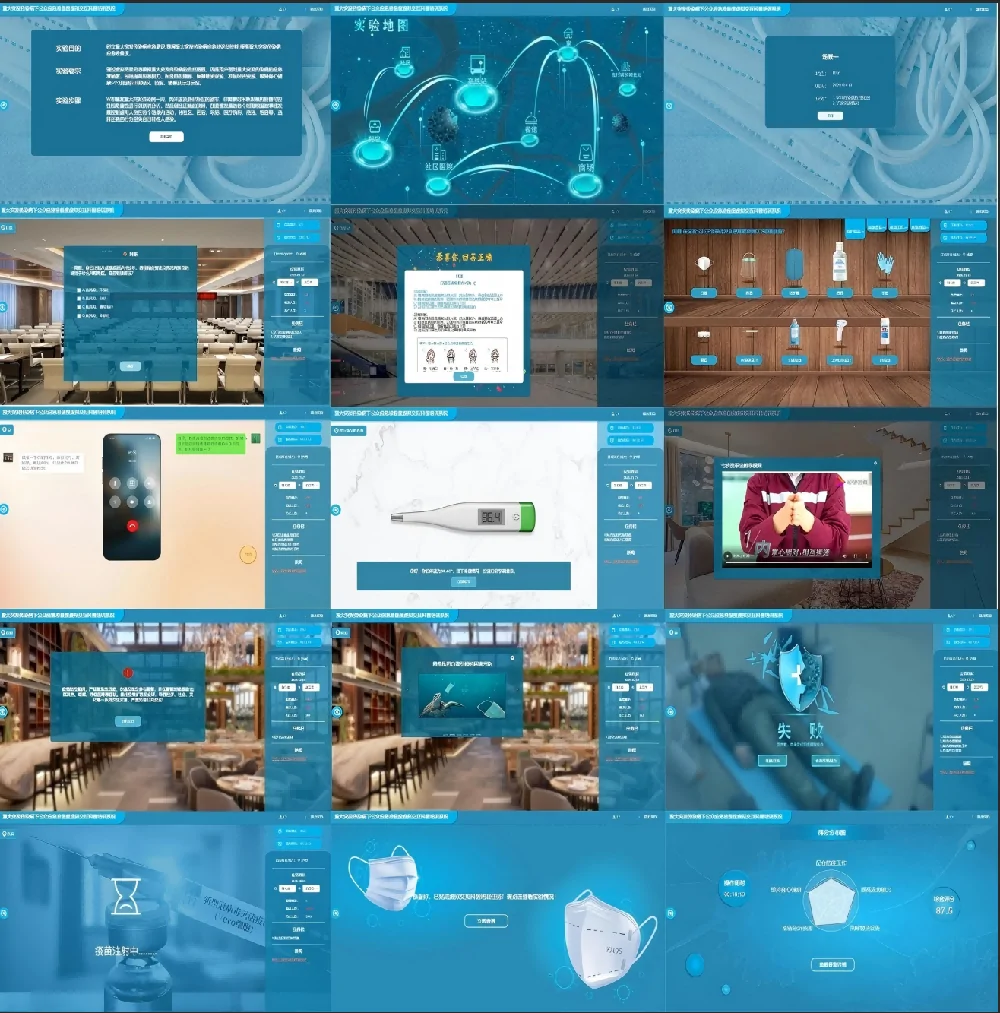
Scene interface of the virtual interactive system “People’s War Against Pandemic.”

#### Control Group

The traditional thematic lecture–based education method was used for a total of 4 weeks. Face-to-face lectures were organized by uniformly trained researchers once a week, with each session lasting for about 40 minutes. Participants were taught the same training knowledge as that provided in the emergency preparedness training content and the knowledge corner. The difference between the 2 groups was that there were no elements such as a storyline, virtual scenes, interaction, or scoring in the thematic lecture–based learning.

#### Intervention Group

Participants were taught the same lectures as the control group each week (conducted by researchers who taught the control group), and then they used the People’s War Against Pandemic for about 40 minutes. Teenagers needed to use the system to complete a storyline task and watch no fewer than 4 articles or videos in the knowledge corner once a week. At least a retest of wrong choices was completed in the intelligent evaluation module. During the implementation of the trial, the researchers monitored participants’ completion status through the user management module, and those who did not finish the task were reminded on the spot or by sending a text message or phone call. This trial was administered by 2 researchers who had systematically studied how to use the system.

#### Ensuring Intervention Fidelity

To ensure the consistency and quality of the lecture intervention across both groups, the following measures were implemented:

- Standardized training: All researchers responsible for delivering the lectures underwent a unified training session. This training covered the exact script, key talking points, and duration of the lecture to minimize presenter variability.
- Use of a structured protocol: A detailed, slide-by-slide presentation protocol was developed and strictly adhered to by all lecturers. This protocol ensured that the core content, examples, and explanations were identical across both the control and intervention groups.
- Monitoring: The principal investigators periodically observed the lecture sessions to ensure compliance with the standardized protocol.

#### Adverse Event Records

During the trial, participants may experience a series of adverse events, such as dizziness, nausea, and eye fatigue [27]. In order to prevent and mitigate these adverse reactions, researchers always paid attention to the health status of the participants and ensured their safety. No adverse events were reported during this study.

### Outcome Measurement

#### Sociodemographic Questionnaire

Sociodemographic data for all participants were gathered using a self-designed questionnaire, including sex, age, ethnicity, family economic income, registered domicile, and self-assessment of infectious diseases.

#### Emergency Preparedness for MEIDs

The emergency preparedness questionnaire for major emerging infectious diseases (EPQ-MEIDs; Multimedia Appendix 1) was developed by our team [1]. The questionnaire included 48 items across 5 dimensions: cooperation with prevention and control work (17 items), preparation of emergency knowledge and coping strategies (16 items), protection of materials and equipment (5 items), adjustment of economic resources (5 items), and maintenance of physical and mental health (5 items). A 5-point Likert scale was used for each item, ranging from 1 (“completely disagree”) to 5 (“completely agree”), with a total score ranging from 48 to 240. The higher the score, the better the emergency preparedness. The Cronbach α coefficient of the questionnaire was 0.953, and the test-retest reliability was 0.900.

#### Infectious Disease–Specific Health Literacy

Infectious Disease–Specific Health Literacy (IDSHL; Multimedia Appendix 2) Scale was developed by Jin et al [28] in 2016 to evaluate the ability of adolescents to acquire and understand the basic knowledge and information related to infectious disease prevention and control. This scale comprises 22 items across 4 dimensions: basic knowledge and concepts of infectious diseases, prevention of infectious diseases, management and treatment of infectious diseases, and identification of pathogens and sources of infection. It consists of 10 judgment questions and 12 single-choice questions. The difficulty coefficients of the 22 questions ranged from 1.19 to 4.08, and the difficulty coefficient for each question was used as the score for a correct answer. Incorrect answers received no score, whereas correct answers received the corresponding score. The full score was 38.62 points. Higher scores signify a stronger sense of infectious disease health literacy among adolescents. The Cronbach α coefficient of the scale was 0.832.

#### System Usability

The System Usability Scale (SUS; Multimedia Appendix 3) was compiled by John Brooke [29] in 1986 and translated into the Chinese version by Wang [30], which was used to assess the overall usability of the system [31]. A 5-point Likert scale was used to evaluate the 10 items. Among them, the odd-numbered items were positively scored, and the actual score was the “original score minus 1.” The even-numbered items were reverse-scored, and the actual score was “5 minus the original score.” If an item was not filled in, the original score was given by default as 3 points, and the actual score was 2 points. The final total score was the sum of all items multiplied by 2.5, with a total score of 100 points. Higher scores indicate better usability of the system. The Cronbach α coefficient of the scale was 0.900.

### Data Collection

The sociodemographic questionnaire, the EPQ-MEIDs, and the IDSHL Scale were administered at baseline (T1), 2 weeks of intervention (T2), 4 weeks of intervention (T3), and 2 weeks after the intervention (T4). The SUS was administered at 2 weeks of intervention (T3). The baseline data of the participants were collected after the participants or their guardians signed the informed consent. The researchers who collected the data received standardized training.

### Statistical Analysis

The data were analyzed using SPSS (version 26.0, IBM Corp). The Shapiro-Wilk test was used to assess the normality of the distribution and the homogeneity of variances. Frequencies, percentages, means, and SDs were used for statistical analysis. The 2-tailed *t* test, Mann-Whitney *U* test, or chi-square test, as applicable, was used to compare the demographic characteristics of the participants. The overall comparison of outcome indicators between the 2 groups across 4 time points was conducted using repeated-measures ANOVA, and an independent-samples *t* test was used to test baseline differences in each index. Significance was defined as *P*<.05.

### Randomization

A random number table was generated using Excel 2016, and the numbers were divided into the intervention and control groups with an allocation ratio of 1:1. The random numbers were then placed in opaque, sealed envelopes and kept by people who did not participate in the study. Therefore, the allocation sequence was concealed from both participants and researchers at the time of enrollment. For each eligible adolescent, the researcher randomly selected one of the envelopes (for each envelope selected by the researcher, the total number of envelopes would be reduced by 1) and allocated them to the intervention or control group. Participants used a code instead of their real names to conceal allocation information, so that the researchers could not know the grouping of the participants. However, due to the nature of the interventions (virtual interactive system vs traditional lecture), participants became aware of their group assignments upon commencement of the intervention.

### Blinding

Given the overtly different nature of the 2 interventions, blinding of the participants and the trial implementers was not feasible. Therefore, only the data collectors and analysts were blinded; that is, the data collectors were not clear about the grouping, and the data analysts obtained only data marked as A and B groups.

### Preventing Contamination

The intervention sessions for the control and intervention groups were scheduled at different times (eg, on different days or in different time slots on the same day) to minimize opportunities for immediate interaction and discussion about the group-specific activities. All participants were explicitly instructed at the beginning of the study not to discuss the specific content of their training sessions (especially the virtual interactive system) with students from other groups until the entire study was completed. The importance of this for the validity of the research was emphasized. The researchers managing the virtual interactive system for the intervention group were different from those primarily involved in data collection, further reducing the risk of inadvertently mentioning the system to control group participants.

### Ethical Considerations

The trial was conducted in accordance with the Declaration of Helsinki, approved by the Human Research Ethics Committee of the Affiliated Hospital of Southwest Medical University (KY2022001), and registered with the Chinese Clinical Trials Registry (ChiCTR2200060919). Once selected, the participants signed an informed written consent form, including explanations about the study’s purpose and research participation conditions. All information shared by the participants was kept confidential. A gift was given to participants after completing daily tasks and questionnaires every week, and participants who participated in the whole study were rewarded with 50 RMB (US $7.5).

## Results

### Participant Flow

A total of 120 participants were recruited and randomly allocated to the intervention group (n=60) and the control group (n=60). All 120 participants completed the 6-week assessments. Ultimately, the analysis comprised 120 participants. Figure 3 shows the CONSORT flowchart of the recruitment process.

**Figure 3.**
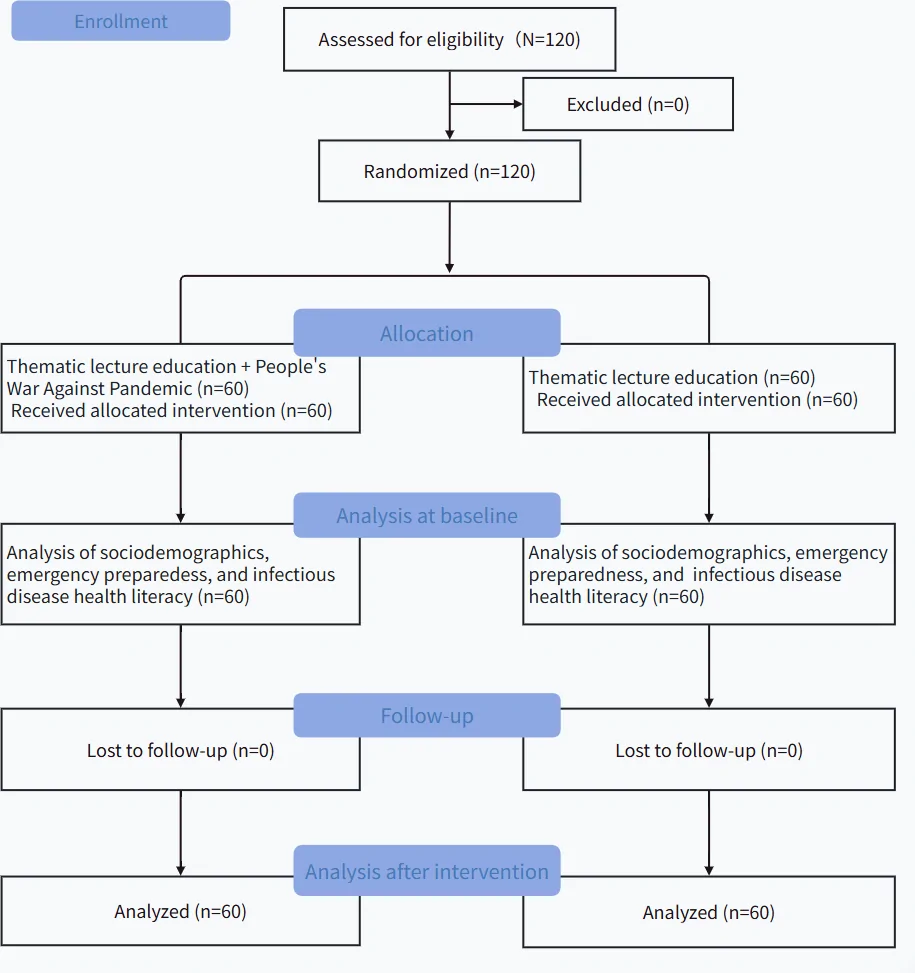
CONSORT flow diagram of participant recruitment, allocation, and follow-up in a randomized controlled trial evaluating a virtual interactive system for emergency preparedness among adolescents in Luzhou, China (2023).

### Demographic Characteristics

The demographic information of the participants is presented in Table 1. The mean age was 17.60 (SD 0.56) years for the intervention group and 17.65 (SD 0.52) years for the control group. The following variables did not demonstrate significant differences between the 2 groups: sex, age, ethnicity, family economic income, or place of residence, indicating that the groups were homogeneous.

**Table 1.** Baseline demographic and clinical characteristics of the study participants by group (intervention vs control) in a randomized controlled trial of a virtual interactive system for adolescent emergency preparedness in Luzhou, China.

Characteristic	Intervention (n=60)	Control (n=60)	*t* test (*df*)/Chi-square test (*df*)	*P* value
Sex, n (%)			2.157^a^ (1)	.14
Male	9 (15)	4 (7)		
Female	51 (85)	56 (93)		
Age (y), mean (SD)	17.60 (0.56)	17.65 (0.52)	−0.510^b^ (118)	.61
Ethnic, n (%)			0.152^a^ (1)	.70
Han nationality	56 (93)	57 (95)		
Others	4 (7)	3 (5)		
Registered domicile, n (%)			0.833^a^ (1)	.36
Rural	50 (83)	46 (77)		
Towns	10 (17)	14 (23)		
Religious belief, n(%)			Fisher exact	.44
Yes	2 (3)	5 (8)	—^c^	
No	58 (97)	55 (92)	—	
Monthly household income, n (%)			Fisher exact	.67
≤1000	3 (5)	1 (2)	—	
1001-4000	27 (45)	33 (55)	—	
4001-7000	22 (37)	17 (28)	—	
7001-10,000	7 (12)	7 (12)	—	
>10,000	1 (1)	2 (3)	—	
Do you know about infectious diseases, n (%)			0.120^a^ (1)	.73
Yes	55 (92)	56 (93)		
No	5 (8)	4 (7)		
Ways, n (%)				
Television	35 (58)	41 (68)	1.292 (1)	.26
Newspapers	5 (8)	8 (13)	0.963 (1)	.33
Network	51 (85)	54 (90)	0.686 (1)	.41
WeChat	36 (60)	34 (57)	0.137 (1)	.71
Microblog	38 (63)	36 (60)	0.141 (1)	.71
School education	46 (77)	47 (78)	0.048 (1)	.83
Medical staff	28 (47)	29 (48)	0.033 (1)	.86
Friends or relatives	35 (58)	37 (62)	0.139 (1)	.71
Other	0 (0)	0 (0)	—	—

a *χ*2 tests.

b *t* tests.

c Not applicable.

### Changes in Emergency Preparedness Scores Over Time

As shown in Table 2, at baseline, there was no significant difference in the average score of emergency preparedness for MEIDs between the 2 groups (*P*=.55). However, the average emergency preparedness score for the intervention group at T2, T3, and T4 was higher than that of the control group. The difference was statistically significant (*P*<.001).

**Table 2.** Comparison of emergency preparedness questionnaire for major emerging infectious diseases (EPQ-MEIDs) scores between the intervention and control groups across the 4 assessment time points (baseline, 2 wk, 4 wk, and 2-wk follow-up)^a^.

Groups	T1	T2	T3	T4
Control, mean (SD)	182.55 (12.69)	184.13 (11.29)	194.52 (10.65)	198.87 (16.75)
Intervention, mean (SD)	184.13 (16.25)	194.28 (11.98)	221.00 (15.25)	220.32 (11.41)
*t* test (*df*)	0.595 (118)	4.777 (118)	11.029 (118)	8.198 (118)
*P* value	.55	<.001	<.001	<.001
Cohen *d*	0.109	0.872	2.014	1.497

a Repeated-measures ANOVA results for EPQ-MEIDs scores: *F *statistic estimate, *P* value, and *ηp*2 for group are 109.186 (1, 118), <.001, and 0.481; for time (overall) are 129.692 (2.786, 328.790), <.001, and 0.524; and for time × group interaction are 23.476 (2.786, 328.790), <.001, and 0.166, respectively.

The data were tested for normality and met this assumption. However, Mauchly test of sphericity indicated a violation of the sphericity assumption (*W*=0.887; *P*<.05). Therefore, we applied the Greenhouse-Geisser correction to adjust the degrees of freedom and reported the corrected values. The results were as follows—group: *F*_1,118_=109.186 (*P*<.001; *ηp*²=0.481), time: *F*_2.786,328.790_=129.692 (*P*<.001; *ηp*²=0.524), and time × group interaction: *F*_2.786,328.790_=23.476 (*P<*.001; *ηp*²=0.166), indicating that the interaction effect between time and group on emergency preparedness scores was significant.

The results of the simple effects analysis (Table 3) showed that the emergency preparedness scores of the intervention group differed significantly over time (*F*_3,177_=117.890; *P*<.001; *ηp*²=0.666). This demonstrated that the virtual interactive system training had a substantial effect, which improved markedly from baseline to week 4 and was maintained at the 2-week follow-up. Although the emergency preparedness scores of the control group also differed significantly across time (*F*_2.508,147.974_=26.136; *P*<.001; *ηp*²=0.307), there was no significant difference between T2 and T1 (*P*=.97, which is >.05), indicating that the emergency preparedness scores increased slowly in the control group, and that the effect was weaker than that in the intervention group.

**Table 3.** Simple effects analysis of emergency preparedness questionnaire for major emerging infectious diseases (EPQ-MEIDs) scores in the intervention and control groups over time.

Groups across time	Mean difference (i − j)	*F* test (*df*)	*P* value	*ηp* ^2^
Between-group comparisons				
Intervention vs control (T1^a^)	1.583	0.354 (1, 118)	.55	0.003
Intervention vs control (T2^b^)	10.150	22.818 (1, 118)	<.001	0.162
Intervention vs control (T3^c^)	26.483	121.642 (1, 118)	<.001	0.508
Intervention vs control (T4^d^)	21.450	67.210 (1, 118)	<.001	0.363
Within-group comparisons				
Intervention group				
Time (overall)	—^e^	117.890 (3, 177)	<.001	0.666
T2 vs T1	10.150	19.938 (1, 59)	<.001	0.253
T3 vs T1	36.867	209.748 (1, 59)	<.001	0.780
T4 vs T1	36.183	217.970 (1, 59)	<.001	0.787
T3 vs T2	26.717	123.461 (1, 59)	<.001	0.677
Control group				
Time (overall)	—	26.136 (2.508, 147.974)	<.001	0.307
T2 vs T1	1.583	0.905 (1, 59)	.97	0.015
T3 vs T1	11.967	34.248 (1, 59)	<.001	0.367
T4 vs T1	16.317	40.972 (1, 59)	<.001	0.410
T3 vs T2	10.383	33.130 (1, 59)	.003	0.360

a T1: baseline.

b T2: 2 weeks of intervention.

c T3: 4 weeks of intervention.

d T4: 2 weeks after intervention.

e Not applicable.

### Changes in Health Literacy Scores Over Time

As shown in Table 4, at baseline, there was no significant difference in the average score of health literacy between the 2 groups (*P*=.40). However, the average health literacy scores for the intervention group at T2, T3, and T4 were significantly higher than those of the control group; the difference was statistically significant (*P=*.01*, P*<.001, and *P*<.001, respectively).

**Table 4.** Comparison of Infectious Disease–Specific Health Literacy (IDSHL) scores between the intervention and control groups across 4 assessment time points^a^.

Groups	T1^b^	T2^c^	T3^d^	T4^e^
Control, mean (SD)	22.12 (5.19)	24.35 (6.70)	25.90 (5.85)	25.84 (5.97)
Intervention, mean (SD)	21.24 (6.09)	27.10 (4.80)	30.19 (6.20)	29.80 (4.70)
*t* test (*df*)	−0.848 (118)	2.586 (118)	3.912 (118)	4.038 (118)
*P* value	.40	<.05	<.001	<.001
Cohen *d*	−0.155	0.472	0.714	0.737

a The independent-samples 2-tailed *t* test was used for between-group comparisons at each time point. The repeated-measures ANOVA assessed the overall effects across time and between groups. Repeated-measures ANOVA results for emergency preparedness questionnaire for major emerging infectious diseases (EPQ-MEIDs) scores: *F *statistic estimate, *P* value, and *ηp*2 for group are 18.480 (1, 118), <.001, and 0.135; for time (overall) are 35.047 (3, 354), <.001, and 0.229; and for time × group interaction are 5.652 (3, 354), <.05, and 0.046, respectively.

b T1: baseline.

c T2: 2 weeks of intervention.

d T3: 4 weeks of intervention.

e T4: 2 weeks after intervention.

The 2 sets of data were tested for normality, and the data satisfied Mauchly sphericity test (*W*=0.943; *P*>.05). The results were as follows—group: *F*_1,118_=18.480 (*P*<.001; *ηp*²=0.135), time: *F*_3,354_=35.047 (*P*<.001; *ηp*²=0.229), time × group interaction: *F*_3,354_=5.652 (*P*<.05; *ηp*²=0.046), indicating that the interaction effect between time and group on health literacy scores was significant.

The results of the simple effects analysis (Table 5) showed that the health literacy scores of the intervention group differed significantly across time (*F*_2.587,152.620_=34.896; *P*<.001; *ηp*²=0.372), indicating that the virtual interactive system training had a substantial effect on improving infectious disease-related health literacy. The scores increased markedly from baseline to week 4 and were maintained at the 2-week follow-up. For the control group, health literacy scores also differed significantly across time (*F*_3,177_=6.235; *P*<.001; *ηp*²=0.096). Although there was a significant increase from T1 to T2 (*P*=.04), no significant difference was found between T3 and T2 (*P*=.16), indicating that the effect of traditional lectures on improving adolescent health literacy was relatively slow and weaker than that of the virtual interactive system.

**Table 5.** Simple effects analysis of Infectious Disease–Specific Health Literacy (IDSHL) scores for the intervention and control groups over time.

Groups	Mean difference	*F* test (*df*)	*P* value	*ηp* ^2^
Between-group comparisons				
Intervention vs control (T1^a^)	−0.876	0.720 (1, 118)	.40	0.006
Intervention vs control (T2^b^)	2.752	6.690 (1, 118)	.01	0.054
Intervention vs control (T3^c^)	4.303	15.303 (1, 118)	<.001	0.115
Intervention vs control (T4^d^)	3.961	16.305 (1, 118)	<.001	0.121
Within-group comparisons				
Intervention group				
Time (overall)	—^e^	34.896 (2.587, 152.620)	<.001	0.372
T2 vs T1	5.859	36.100 (1, 59)	<.001	0.380
T3 vs T1	8.955	53.025 (1, 59)	<.001	0.473
T4 vs T1	8.561	71.025 (1, 59)	<.001	0.546
T3 vs T2	3.096	11.114 (1, 59)	.001	0.159
Control group				
Time (overall)	—	6.235 (3, 177)	<.001	0.096
T2 vs T1	2.231	4.584 (1, 59)	.04	0.072
T3 vs T1	3.776	15.268 (1, 59)	<.001	0.206
T4 vs T1	3.724	14.403 (1, 59)	<.001	0.196
T3 vs T2	1.544	1.981 (1, 59)	.16	0.032

a T1: baseline.

b T2: 2 weeks of intervention.

c T3: 4 weeks of intervention.

d T4: 2 weeks after the intervention.

e Not applicable.

### Availability of the Virtual Interactive System

The total average usability evaluation score of the virtual interaction system by adolescents was 85.75, as shown in Table 6. The item with the highest score on the scale was item 1, “I will be willing to use (login/access) this system frequently,” and the item with the lowest score was item 6, “I thought there was too much inconsistency in this system.”

**Table 6.** Availability score of the virtual interactive system (n=60)^a^.

Items	Mean (SD)
1. I will be willing to use (login/access) this system frequently.	3.833 (0.376)
2. I think this system is too complex.	3.500 (0.624)
3. I think this system is easy to use.	3.700 (0.530)
4. I think I need the help of technical staff to use this system.	3.550 (0.62)
5. I think the various modules of this system are integrated very well.	3.017 (0.596)
6. I thought there was too much inconsistency in this system.	2.650 (0.606)
7. I think most people can learn to use this system quickly.	3.733 (0.446)
8. I think this system is troublesome to use.	2.917 (0.530)
9. I am confident to use this system skillfully.	3.733 (0.634)
10. In order to operate the system, I need to learn a lot of new knowledge.	3.667 (0.475)
Total score	85.750 (5.152)

a Values were transformed contribution scores per standard System Usability Scale (SUS) scoring. Odd items (positive): contribution = (raw score – 1); even items (negative): contribution = (5 – raw score). Sum of contributions × 2.5 = total SUS score.

## Discussion

### Virtual Interaction Systems Could Enhance the Emergency Preparedness of Adolescents for MEID Outbreaks

Historical pandemics have demonstrated that adolescents are not only potential vectors for pathogen transmission but also a critical force in epidemic prevention and control [32]. The participation and cooperation of adolescents in epidemic prevention and control are key factors in curbing the spread of the epidemic [33]. Although health departments and government agencies play a central role in formulating response strategies and providing medical services, these efforts are likely to be in vain without the active response of adolescents. Therefore, effective emergency preparedness for adolescents can significantly reduce the risk of transmission during MEID outbreaks [34].

The results of this study suggest that virtual interactive system training can enhance adolescents’ emergency preparedness more effectively than traditional lectures alone. This finding aligns with previous research conducted among nursing students and health care workers, which also found that virtual simulation training significantly improved emergency response skills and knowledge retention [19,35,36]. While a direct statistical comparison is not feasible due to differing outcome measures, the magnitude of improvement observed in our adolescent population appears substantial. For instance, the intervention group’s emergency preparedness score increased by approximately 20% from baseline to the end of the intervention, a rate of improvement that is comparable to, and in some cases exceeds, the effects reported in studies with professional groups using similar simulation-based training [37]. This suggests that narrative-driven virtual interactive systems could achieve meaningful learning outcomes in a general adolescent population, not just among motivated health care trainees.

Our study extends this evidence by demonstrating that similar benefits can be achieved in a nonprofessional, adolescent population using a narrative-based, publicly-focused platform, a finding corroborated by other research highlighting the efficacy of VR in creating engaging learning environments without physical space restrictions [38]. This can be attributed to the interaction of various factors. On the one hand, “People’s War Against Pandemic” provided rich environmental stimulation through virtual pandemic life scenarios. Moreover, its multidomain task modes offer more targeted stimulation to participants, granting them comprehensive sensory experiences. This helps them familiarize themselves with and master quarantine norms as well as basic self-help and mutual rescue skills. On the other hand, the effectiveness of the system can be explained by the theory of embodied cognition. Unlike passive learning in lectures, our virtual system provides a highly immersive environment that promotes embodied learning. Users learn by doing and making decisions within a simulated context. This approach has been shown to enhance situational awareness, deepen cognitive understanding, and facilitate the transfer of knowledge into behavioral intention and change [39]. Furthermore, the immersive nature of VR and augmented reality systems has been specifically linked to increased user engagement and improved comprehension levels in educational interventions [38], while also being effective in developing empathy through perspective-taking, which could be crucial in fostering compliance with public health measures that protect others.

In this study, after just 2 weeks of training in the intervention group, the emergency preparedness score was close to that of the control group after 4 weeks of education. This may be because the comprehension of scientific knowledge about infectious diseases in lectures was difficult to understand, requiring sustained stimulation over a longer period before effects began to appear [40]. It is evident from this that “People’s War Against Pandemic” could enhance adolescents’ emergency preparedness more rapidly compared to lectures, highlighting its application advantages.

However, it is important to interpret these findings with caution. In this study design, the intervention group received approximately 80 minutes of total educational exposure (a 40-min lecture + 40-min virtual system use), while the control group received only 40 minutes of lecture-based education. Therefore, the observed superior outcomes in the intervention group may be partially or wholly attributable to the increased time-on-task rather than any inherent superiority of the virtual interactive system as a pedagogical modality. This confounding variable precludes a direct comparison of the relative efficacy of the 2 instructional methods under time-equivalent conditions.

### Virtual Interaction Systems Could Enhance IDSHL Among Adolescents

Studies have indicated that enhancing IDSHL among adolescents can help alleviate their panic and maintain social stability [41]. Therefore, IDSHL is regarded as one of the critical indicators for evaluating the effectiveness of health education on infectious diseases [42].

The results of this study found that traditional lecture education had a slower effect, while “People’s War Against Pandemic” boasts a more significant advantage in enhancing adolescents’ IDSHL. Jacque et al [43] integrated infectious disease knowledge into a school curriculum and found improvements in health literacy, albeit to a lesser extent than observed in our study. This difference in efficacy may be attributed to the pedagogical approach. While their study used a traditional classroom-based curriculum, our interactive system provided experiential learning through simulation, which is posited to lead to deeper cognitive processing and engagement [21]. The meta-analysis further supports that the impact of virtual experiments on learning effectiveness is significant and can be influenced by factors such as the design and implementation of the virtual experience [21], which aligns with our system’s thoughtful integration of multiple interactive modules. First, most traditional lectures are static, graphic-and-text-based education, lacking interaction [44]. However, “People’s War Against Pandemic” provides opportunities for real-time feedback and dynamic participation, enhancing the learning interest and enthusiasm of adolescents and promoting their active involvement in the practice of infectious disease prevention and control. Second, the storyline and task design of “People’s War Against Pandemic” are continuous and logical, which can progressively enhance adolescents’ health literacy regarding infectious diseases, from basic theoretical knowledge to specific operational practice. Finally, neuroscientists believe that virtual systems involving task failures will lead to emotional arousal, promoting an increase in noradrenaline levels in the amygdala, which helps reinforce the process of memory formation and stabilization. This is beneficial for the retention of knowledge [45].

Furthermore, the observed plateauing of scores at follow-up warrants careful consideration. While common in educational and behavioral interventions, as the initial intensity of the training fades, it highlights the potential need for “booster” sessions or the integration of such virtual training into regular school health curricula to sustain its effects. This pattern underscores that emergency preparedness is not a one-time educational event but a skill that may require periodic reinforcement. Future studies should prioritize long-term follow-up assessments (eg, 6 mo, 1 y) and, where ethically and logistically feasible, use behavioral measures or simulations closer to real-world scenarios to evaluate the true translational impact of the virtual interactive system on actual emergency response behaviors.

### The Usability of Virtual Interaction Systems Was Relatively High, Capable of Meeting the Core Needs of Adolescents

The average SUS score in this study was 85.75, indicating that this system had excellent usability [46]. The results showed that item 1 scored the highest, followed by items 7 and 9, suggesting that the system’s design was intuitive, the user interface was friendly, and the operation process was straightforward, fulfilling users’ needs and expectations. Salamah et al [47] pointed out that user satisfaction with a system is a key factor driving their intention to revisit. The innovation of the system, the practicality of the content, and the ease of use are the 3 direct factors affecting user satisfaction. Item 6 (“I thought there was too much inconsistency in this system”) received the lowest transformed contribution score, identifying perceived internal consistency as the relatively weakest aspect of the system and the most useful target for future refinement. Nonetheless, the overall SUS score of 85.75 indicates excellent usability.

### Limitations

First, this study was conducted at a single vocational school in Luzhou, Sichuan, which may limit the generalizability of the findings to adolescents in other regions or educational contexts. However, following the Spatial Statistic Trinity (SST) framework, we have provided comprehensive details to enhance transferability.

Regarding population characteristics, our participants were adolescents aged 16 to 19 years (mean 17.60, SD 0.56) from a lower-middle-income region of China, with demographic profiles typical of vocational school populations in Southwest China (eg, 107/120, 89% female participants; 96/120, 80% rural areas). These characteristics are relevant to similar socioeconomic and educational settings.

The study used a randomized controlled design with concealed allocation, standardized intervention protocols, and complete follow-up (0% attrition), which strengthens internal validity and supports replication in analogous study contexts.

For estimation methods, the study used repeated-measures ANOVA with Greenhouse-Geisser correction where appropriate, reported effect sizes (partial *η*²), and provided raw data for key outcomes. Transparent reporting of these methods allows researchers to compare our estimates with those from their own settings.

The interaction of these 3 elements suggests that our findings are likely transferable to similar adolescent populations in comparable educational settings (eg, vocational schools in other Chinese cities with similar sociodemographic profiles). However, transferability to dissimilar populations (eg, younger adolescents, different cultural contexts, or nonvocational school settings) requires further empirical testing.

Although the sample size was statistically adequate for this RCT, a larger and more diverse sample would further improve the representativeness of the results. Second, the intervention and follow-up periods were relatively short, which precludes conclusions regarding the long-term efficacy of the virtual interactive system. Future multicenter studies with extended follow-up durations are recommended to validate these preliminary findings and further explore the transferability of the intervention across diverse populations and settings.

### Conclusions

The findings of this study demonstrate that, compared with traditional lectures alone, the addition of the “People’s War Against Pandemic” virtual interactive system to lecture-based education led to significantly greater improvements in self-reported emergency preparedness and infectious disease health literacy among adolescents. These results suggest that the system effectively enhanced participants’ perceived readiness and knowledge regarding MEIDs. However, as the outcomes were measured using self-report questionnaires, conclusions regarding actual behavioral changes or objective decision-making skills cannot be drawn from this study. Future research should incorporate objective behavioral assessments or simulated emergency scenarios to evaluate the translation of these self-reported improvements into real-world response capabilities. Due to the unequal total educational time between groups, the observed effects may reflect increased learning time rather than the inherent superiority of the virtual modality. Future research using time-controlled designs is needed to isolate the specific contribution of virtual interactive training. The system has a good user experience and a certain usability, which was worthy of promotion and use.

## Supplementary material

10.2196/67595 Multimedia Appendix 1 Emergency preparedness questionnaire for major emerging infectious diseases.

10.2196/67595 Multimedia Appendix 2 Infectious Disease–Specific Health Literacy Scale and difficulty coefficients.

10.2196/67595 Multimedia Appendix 3 System Usability Scale.

10.2196/67595 Checklist 1 CONSORT checklist.

## Abbreviations

CONSORT: Consolidated Standards of Reporting Trials
EPQ-MEIDs: emergency preparedness questionnaire for major emerging infectious diseases
EVD: Ebola virus disease
IDSHL: Infectious Disease–Specific Health Literacy
MEIDs: major emerging infectious diseases
MERS: Middle East respiratory syndrome
NHS: National Health Service
RCT: randomized controlled trial
SST: Spatial Statistic Trinity
SUS: System Usability Scale
VR: virtual reality
