# Supplementary material for: Effects of a Virtual Interactive System (People’s War Against Pandemic) on Emergency Preparedness for Major Emerging Infectious Diseases Among Adolescents: Randomized Controlled Trial
Source: JMIR Serious Games. 2026 Oct 26;14:e67595. doi: 10.2196/67595 (PMC13705210; doi:10.2196/67595)
Supplement: Supplementary file 2 [file games-v14-e67595-s001.docx]

**重大突发传染病事件下公众应急准备度自评问卷**

| 维度 | 条目 | 完全不同意(1) | 不同意(2) | 一般(3) | 同意(4) | 完全同意(5) |
| --- | --- | --- | --- | --- | --- | --- |
| 配合防控工作 | 1.我能配合政府颁布的疫情管控政策、政令 |  |  |  |  |  |
|  | 2.我能配合落实疾病预防控制机构疫情防控方案、措施 |  |  |  |  |  |
|  | 3.我能配合医疗机构疫情期间诊疗工作 |  |  |  |  |  |
|  | 4.我能配合所在单位、属地疫情管控工作 |  |  |  |  |  |
|  | 5.我能配合交通站点疫情防控工作 |  |  |  |  |  |
|  | 6.我能配合出入境口岸疫情防控工作 |  |  |  |  |  |
|  | 7.我会主动向所到社区动态报备个人状况 |  |  |  |  |  |
|  | 8.我能配合疫情期间个人相关信息收集、报告 |  |  |  |  |  |
|  | 9.我能严格服从各种隔离相关管控要求 |  |  |  |  |  |
|  | 10.我能理解和支持有限条件下疫情防控工作中可能存在的疏漏 |  |  |  |  |  |
|  | 11.我能严格服从消毒防控要求 |  |  |  |  |  |
|  | 12.我了解个人配合防控工作的益处 |  |  |  |  |  |
|  | 13.我能配合公共场所疫情管控工作 |  |  |  |  |  |
|  | 14.我会积极参与疫情防控志愿服务 |  |  |  |  |  |
|  | 15.我不编造、不传播虚假疫情信息 |  |  |  |  |  |
|  | 16.我不瞒报、不伪造个人相关信息 |  |  |  |  |  |
|  | 17.我不阻碍工作人员执行公务 |  |  |  |  |  |
| 提高应急能力 | 18.我会学习传染病基本知识 |  |  |  |  |  |
|  | 19.我会学习传染病危害性 |  |  |  |  |  |
|  | 20.我会学习传染病防控措施 |  |  |  |  |  |
|  | 21.我关注权威机构部门发布的疫情相关信息 |  |  |  |  |  |
|  | 22.我能准确判断自身感染的可能性 |  |  |  |  |  |
|  | 23.我能关注疫情的动态变化 |  |  |  |  |  |
|  | 24.我能保持个人良好卫生习惯 |  |  |  |  |  |
|  | 25.我能保持安全社交距离 |  |  |  |  |  |
|  | 26.我能做好外出、居家消毒工作 |  |  |  |  |  |
|  | 27.我能规范穿戴防护用具 |  |  |  |  |  |
|  | 28.我能主动做好家庭成员与个人的健康监测 |  |  |  |  |  |
|  | 29.我能积极接种相应疫苗 |  |  |  |  |  |
|  | 30.我会预先了解可提供帮助机构的类目 |  |  |  |  |  |
|  | 31.我熟知各类机构求助电话 |  |  |  |  |  |
|  | 32.我熟知寻求帮助的流程 |  |  |  |  |  |
|  | 33.我了解紧急就医流程 |  |  |  |  |  |
| 保障物资设备 | 34.我会购买足量的家庭防护用品，如口罩、一次性手套等 |  |  |  |  |  |
|  | 35.我能保证个人电话等通讯方式畅通 |  |  |  |  |  |
|  | 36.我会购买适量应急生活物资，如粮油米面 |  |  |  |  |  |
|  | 37.我会购买适量应急药品，如退热药 |  |  |  |  |  |
|  | 38.我会购买适量应急工具，如电源设备 |  |  |  |  |  |
| 准备经济资源 | 39.我能预估疫情对个人经济收入带来的损失 |  |  |  |  |  |
|  | 40.我能预估防疫物资整体支出 |  |  |  |  |  |
|  | 41.我能预估个人因疫情而产生的医疗支出 |  |  |  |  |  |
|  | 42.我能预估个人除防疫与医疗支出外的其它支出，如生活支出 |  |  |  |  |  |
|  | 43.我能根据收入与支出对经济资源进行相应调整 |  |  |  |  |  |
| 维持身心健康 | 44.在疫情期间我能规律作息 |  |  |  |  |  |
|  | 45.在疫情期间我能坚持锻炼身体 |  |  |  |  |  |
|  | 46.在疫情期间我能保证健康饮食 |  |  |  |  |  |
|  | 47.我对疫情有正确的认知，可以减少不当的恐慌 |  |  |  |  |  |
|  | 48.我能合理管控情绪，必要时会寻求心理救助 |  |  |  |  |  |

Emergency preparedness questionnaire for MEIDs (EPQ-MEIDs)

| dimensions | items | Completely Disagree(1) | Disagree(2) | Neutral(3) | Agree(4) | Completely Agree(5) |
| --- | --- | --- | --- | --- | --- | --- |
| Cooperating with prevention and control work | A1. I comply with government policies and decrees on epidemic prevention and control |  |  |  |  |  |
|  | A2. I am willing to cooperate with disease prevention programs |  |  |  |  |  |
|  | A3. I comply with the diagnosis and treatment procedures of medical institutions during the epidemic. |  |  |  |  |  |
|  | A4. I am willing to cooperate with the epidemic situation control work in the place where the individual is located |  |  |  |  |  |
|  | A5. I am willing to cooperate with epidemic prevention and control at traffic stations |  |  |  |  |  |
|  | A6. I am willing to cooperate with epidemic prevention and control at entry-exit ports |  |  |  |  |  |
|  | A7. I am willing to proactively report my personal status to the community I arrive in |  |  |  |  |  |
|  | A8. I am willing to cooperate with the collection and report of relevant personal information during epidemic situation |  |  |  |  |  |
|  | A9. I am willing to strictly comply with various control requirements related to isolation |  |  |  |  |  |
|  | A10. I am willing to understand and support possible omissions in epidemic prevention and control under limited conditions |  |  |  |  |  |
|  | A11. I strictly obey the disinfection prevention and control requirements |  |  |  |  |  |
|  | A12. I understand the benefits of individual cooperation in prevention and control |  |  |  |  |  |
|  | A13. I am willing to cooperate with epidemic control in public places |  |  |  |  |  |
|  | A14. I actively participate in voluntary service for epidemic prevention and control |  |  |  |  |  |
|  | A15. I do not fabricate or disseminate false epidemic information. |  |  |  |  |  |
|  | A16. I do not conceal or forge personal information |  |  |  |  |  |
|  | A17. I do not hinder the staff to perform official duties |  |  |  |  |  |
| Preparing knowledge and coping strategies | B18. I am willing to learn basic knowledge of infectious diseases |  |  |  |  |  |
|  | B19. I am willing to learn about the dangers of infectious diseases |  |  |  |  |  |
|  | B20. I am willing to learn about the prevention and control measures of infectious disease |  |  |  |  |  |
|  | B21. I am willing to pay attention to the information related to epidemic situation released by authorities and departments |  |  |  |  |  |
|  | B22. I accurately determine the possibility of self-infection |  |  |  |  |  |
|  | B23. I am willing to pay attention to dynamic changes of epidemic |  |  |  |  |  |
|  | B24. I am willing to maintain good personal hygiene |  |  |  |  |  |
|  | B25. I am willing to maintain a safe social distance |  |  |  |  |  |
|  | B26. I am willing to do well in disinfection when going out and home |  |  |  |  |  |
|  | B27. I am willing to wear protective equipment correctly |  |  |  |  |  |
|  | B28. I am willing to take the initiative to monitor the health of family members and individuals |  |  |  |  |  |
|  | B29. I actively vaccinate the corresponding vaccine |  |  |  |  |  |
|  | B30. I am willing to know in advance the categories of organizations that can provide assistance |  |  |  |  |  |
|  | B31. I am familiar with the help telephone numbers of various institutions |  |  |  |  |  |
|  | B32. I am familiar with the process of seeking help |  |  |  |  |  |
|  | B33. I am willing to understand emergency medical procedures |  |  |  |  |  |
| Securing supplies and equipment | C34. I am willing to purchase appropriate amount of household protective equipment, such as masks, disposable gloves, etc. |  |  |  |  |  |
|  | C35. I am willing to ensure that personal mobile phones or other means of communication are unobstructed |  |  |  |  |  |
|  | C36. I am willing to purchase appropriate amount of emergency living goods, such as grain, oil and rice |  |  |  |  |  |
|  | C37. I am willing to purchase appropriate amount of emergency medications, such as antipyretics |  |  |  |  |  |
|  | C38. I am willing to purchase appropriate amount of emergency tools, such as power supply equipment |  |  |  |  |  |
| Preparing economic resources | D39. I am willing to estimate the loss of personal economic income caused by the epidemic |  |  |  |  |  |
|  | D40. I am willing to estimate expenditure on purchasing epidemic prevention materials |  |  |  |  |  |
|  | D41. I am willing to estimate personal medical expenses due to the epidemic |  |  |  |  |  |
|  | D42. I am willing to estimate personal expenses other than epidemic prevention and medical expenses, such as living expenses |  |  |  |  |  |
|  | D43. I am willing to adjust economic resources according to income and expenditure |  |  |  |  |  |
| Maintaining physical and mental health | E44. I am willing to maintain regular work and rest during the epidemic |  |  |  |  |  |
|  | E45. I am willing to keep exercising |  |  |  |  |  |
|  | E46. I am willing to ensure a healthy diet |  |  |  |  |  |
|  | E47. I am willing to establish correct awareness of the epidemic and reduce undue panic |  |  |  |  |  |
|  | E48. I am willing to reasonably control personal emotions and seek psychological assistance if necessary |  |  |  |  |  |
