# Supplementary material for: Effects of a Virtual Interactive System (People’s War Against Pandemic) on Emergency Preparedness for Major Emerging Infectious Diseases Among Adolescents: Randomized Controlled Trial
Source: JMIR Serious Games. 2026 Oct 26;14:e67595. doi: 10.2196/67595 (PMC13705210; doi:10.2196/67595)
Supplement: Supplementary file 3 [file games-v14-e67595-s002.docx]

**中国居民传染病健康素养测评量表**

| 您是否同意以下说法？ | 同意 | 不同意 | 不知道 |
| --- | --- | --- | --- |
| 1.得了乙肝（乙型肝炎），要坚持服用抗生素。 | ○ | ○ | ○ |
| 2.吃涮羊肉时，肉的颜色由深变浅说明可以食用了。 | ○ | ○ | ○ |
| 3.乙肝可通过性行为传播。 | ○ | ○ | ○ |
| 4.结核病患者服药治疗两周后，咳嗽咳痰症状消失，体温正常，就说明已经治愈了。 | ○ | ○ | ○ |
| 5.病死家禽经过蒸煮、做熟后食用是完全安全的。 | ○ | ○ | ○ |
| 6.一个人只要感染了艾滋病病毒就能从外表上看出来。 | ○ | ○ | ○ |
| 7.青壮年身体好，是不可能得结核病的。 | ○ | ○ | ○ |
| 8.居家饲养猫、狗，只要不经常外出，也不会咬人，不用给宠物注射疫苗。 | ○ | ○ | ○ |
| 9.自由流动是人的基本权利，不能限制传染病患者自由流动。 | ○ | ○ | ○ |
| 10.患了流感（流行性感冒）就应该尽快注射或服用抗生素。 | ○ | ○ | ○ |

**选择题：以下每个问题只有一个正确答案，请根据自身理解勾选正确答案的序号。**

11. “H1N1”是指

○甲型肝炎	○治疗传染病的一种药物 ○甲型流感	○不知道

12. 从事餐饮行业应到当地卫生部门办理

○用工证	○健康证	○暂住证	○不知道

13. “疫水”是指

○生活污水	○被病原体污染的水体	○传染病疫区的水 ○不知道

14. 小儿服用“糖丸”疫苗是为了预防

○小儿麻痹症 ○肺结核	○麻疹	○不知道

15. “HIV”是指

○一种细菌	○一种病毒	○一种寄生虫	○不知道

16. 您认为出现哪种症状，应该首先怀疑得了手足口病？

○咳嗽、咳痰 ○手、足、口腔粘膜等出现皮疹	○多饮、多尿	○不知道

17. 容易感染手足口病的人群

○学龄前儿童 ○青少年	○中青年	○不知道

18. “疫苗”是指

○治疗传染病的生物制剂	○抗菌素	○预防传染病的生物制剂	○不知道

19. 以下哪种途径会传播乙肝病毒

○共用牙刷	○共用碗筷餐具	○握手	○不知道

20. 如果被猫、狗抓伤/咬伤，很轻，只是破了点皮，您会：

○用清水把伤口冲洗干净，不注射狂犬疫苗

○立即用肥皂和清水清洗伤口，并尽快注射狂犬疫苗

○用民间土方法进行伤口处理

○不知道

21. 被钉子扎伤，需要注射

○“百白破”疫苗	○破伤风疫苗	○狂犬病疫苗	○不知道

22. 蚊子在室内孳生繁殖的场所为

○水沟、水坑、盛水的器皿中	○草丛或树木中	○野外的土壤	○不知道

**Infectious disease specific health literacy(IDSHL)**

| Statement | Agree | Disagree | Don't Know |
| --- | --- | --- | --- |
| 1. For hepatitis B (hepatitis B virus infection), antibiotics should be taken continuously. |  |  |  |
| 2. When eating hot pot lamb, the meat is safe to eat once its color changes from dark to light. |  |  |  |
| 3. Hepatitis B can be transmitted through sexual contact. |  |  |  |
| 4. After two weeks of medication, if a tuberculosis patient no longer has coughing or sputum and has normal body temperature, they are already cured. |  |  |  |
| 5. It is completely safe to eat poultry that died from disease after thoroughly boiling or cooking it. |  |  |  |
| 6. A person infected with HIV can be identified by their appearance. |  |  |  |
| 7. Young and middle-aged adults are physically strong and therefore cannot get tuberculosis. |  |  |  |
| 8. For household pets such as cats and dogs that do not frequently go outside and do not bite people, vaccination is not necessary. |  |  |  |
| 9. Free movement is a basic human right, and the movement of individuals with infectious diseases should not be restricted. |  |  |  |
| 10. When suffering from influenza (flu), one should promptly receive antibiotics via injection or oral administration. |  |  |  |

**Multiple-Choice Questions**
For each of the following questions, there is only one correct answer. Please select the number corresponding to the answer you believe is correct.

11. "H1N1" refers to:
○ Hepatitis A
○ A drug for treating infectious diseases
○ Influenza A
○ Don't know

12. People working in the food service industry should obtain the following document from the local health department:
○ Employment certificate
○ Health certificate
○ Temporary residence permit
○ Don't know

13. "Epidemic water" (Yi Shui) refers to:
○ Domestic wastewater
○ Water contaminated by pathogens
○ Water from epidemic areas
○ Don't know

14. The "sugar pill" vaccine given to children is used to prevent:
○ Poliomyelitis (polio)
○ Tuberculosis
○ Measles
○ Don't know

15. "HIV" refers to:
○ A type of bacterium
○ A virus
○ A parasite
○ Don't know

16. Which symptom should first raise suspicion of hand, foot, and mouth disease?
○ Cough and sputum
○ Rash on hands, feet, and oral mucosa
○ Increased thirst and urination
○ Don't know

17. Which population is most susceptible to hand, foot, and mouth disease?
○ Preschool children
○ Adolescents
○ Young and middle-aged adults
○ Don't know

18. "Vaccine" refers to:
○ Biological preparation for treating infectious diseases
○ Antibiotic
○ Biological preparation for preventing infectious diseases
○ Don't know

19. Which of the following can transmit the hepatitis B virus?
○ Sharing a toothbrush
○ Sharing bowls and chopsticks
○ Handshake
○ Don't know

20. If scratched or bitten by a cat or dog, even if the wound is minor and only breaks the skin, you would:
○ Rinse the wound with clean water only, and do not receive rabies vaccine
○ Immediately wash the wound with soap and water, and promptly receive rabies vaccine
○ Treat the wound using folk remedies
○ Don't know

21. After being punctured by a nail, you should receive:
○ DPT (diphtheria, pertussis, tetanus) vaccine
○ Tetanus vaccine
○ Rabies vaccine
○ Don't know

22. Where do mosquitoes typically breed indoors?
○ Drains, puddles, and containers holding water
○ Grass or trees
○ Outdoor soil
○ Don't know

**Table of Difficulty Coefficients for the 22 Items of the IDSHL Scale**

| **Item No.** | **Difficulty Coefficient** | **Correct Answer** |
| --- | --- | --- |
| 1 | 2.39 | Disagree |
| 2 | 1.96 | Disagree |
| 3 | 4.08 | Agree |
| 4 | 1.57 | Disagree |
| 5 | 1.19 | Disagree |
| 6 | 1.41 | Disagree |
| 7 | 1.22 | Disagree |
| 8 | 1.21 | Disagree |
| 9 | 1.34 | Disagree |
| 10 | 1.89 | Disagree |
| 11 | 1.88 | Influenza A |
| 12 | 1.19 | Health certificate |
| 13 | 2.38 | Water contaminated by pathogens |
| 14 | 1.89 | Poliomyelitis (polio) |
| 15 | 1.97 | A virus |
| 16 | 1.32 | Rash on hands, feet, and oral mucosa |
| 17 | 1.39 | Preschool children |
| 18 | 1.45 | Biological preparation for preventing infectious diseases |
| 19 | 2.72 | Sharing a toothbrush |
| 20 | 1.34 | Immediately wash the wound with soap and water, and promptly receive rabies vaccine |
| 21 | 1.36 | Tetanus vaccine |
| 22 | 1.47 | Drains, puddles, and containers holding water |

Note: Items 1–10 are judgment questions; items 11–22 are multiple‑choice questions. The sum of these coefficients equals the maximum possible score of 38.62.
