# Supplementary material for: Effects of a Virtual Interactive System (People’s War Against Pandemic) on Emergency Preparedness for Major Emerging Infectious Diseases Among Adolescents: Randomized Controlled Trial
Source: JMIR Serious Games. 2026 Oct 26;14:e67595. doi: 10.2196/67595 (PMC13705210; doi:10.2196/67595)
Supplement: Supplementary file 4 [file games-v14-e67595-s003.docx]

**系统可用性量表（System Usability Scale，SUS）**

| 项目 | 非常不同意 | 不同意 | 一般 | 同意 | 非常同意 |
| --- | --- | --- | --- | --- | --- |
| 1.我会愿意经常使用(登陆/访问)这个系统/网站 |  |  |  |  |  |
| 2.我认为这个系统/网站没有必要这么复杂 |  |  |  |  |  |
| 3.我认为这个系统/网站很容易使用 |  |  |  |  |  |
| 4.我认为需要有经验的人帮助我才能使用这一系统/网站 |  |  |  |  |  |
| 5.我认为这个产品/网站的多种功能整合的很好 |  |  |  |  |  |
| 6.我认为这个产品/网站有太多不一致 |  |  |  |  |  |
| 7.我认为大多数人都可以很快学会使用这一产品/网站 |  |  |  |  |  |
| 8.我认为这个产品/网站使用起来很麻烦 |  |  |  |  |  |
| 9.在使用过程中，我感觉很自信 |  |  |  |  |  |
| 10.为了操作这个产品/网站，我需要学习很多东西 |  |  |  |  |  |

**System Usability Scale(SUS)**

| items | Strongly Disagree | Disagree | Neutral | Agree | Strongly Agree |
| --- | --- | --- | --- | --- | --- |
| 1.I will be willing to use ( login / access ) this system frequently. |  |  |  |  |  |
| 2.I think this system is too complex. |  |  |  |  |  |
| 3.I think this system is easy to use. |  |  |  |  |  |
| 4.I think I need the help of technical staff to use this system. |  |  |  |  |  |
| 5.I think the various modules of this system are integrated very well. |  |  |  |  |  |
| 6.I thought there was too much inconsistency in this system. |  |  |  |  |  |
| 7.I think most people can learn to use this system quickly. |  |  |  |  |  |
| 8.I think this system is troublesome to use. |  |  |  |  |  |
| 9.I am confident to use this system skillfully. |  |  |  |  |  |
| 10.In order to operate the system, I need to learn a lot of new knowledge. |  |  |  |  |  |
